# Group reciprocity and the evolution of stereotyping

**DOI:** 10.1098/rspb.2022.1834

**Published:** 2023-01-25

**Authors:** Alexander J. Stewart, Nichola Raihani

**Affiliations:** ^1^ School of Mathematics and Statistics, University of St Andrews, St Andrews KY16 9SS, UK; ^2^ Department of Experimental Psychology, University College London, London, UK

**Keywords:** group reciprocity, judgement bias, cooperation, stereotyping, game theory, cultural evolution

## Abstract

Stereotypes are generalized beliefs about groups of people, which are used to make decisions and judgements about them. Although such heuristics can be useful when decisions must be made quickly, or when information is lacking, they can also serve as the basis for prejudice and discrimination. In this paper, we study the evolution of stereotypes through group reciprocity. We characterize the warmth of a stereotype as the willingness to cooperate with an individual based solely on the identity of the group they belong to. We show that when stereotype groups are large, such group reciprocity is less likely to evolve, and stereotypes tend to be negative. We also show that, even when stereotypes are broadly positive, individuals are often overly pessimistic about the willingness of those they stereotype to cooperate. We then show that the tendency for stereotyping itself to evolve is driven by the costs of cognition, so that more people are stereotyped with greater coarseness as costs increase. Finally we show that extrinsic ‘shocks’, in which the benefits of cooperation are suddenly reduced, can cause stereotype warmth and judgement bias to turn sharply negative, consistent with the view that economic and other crises are drivers of out-group animosity.

## 1. Introduction

Stereotyping, in which a set of characteristics is attributed to all members of an identity group, shapes many human social interactions [1–8]. Such generalizations can reflect or even exacerbate inter-group tensions, leading in the extreme to dehumanization of out-groups [9–11]. More generally, however, stereotyping can be understood as the use of heuristics to guide social decision-making, which can often be a practical necessity [12,13]. If we lack information about an individual’s past behaviour, or if cognitive constraints are present, a combination of positive and negative stereotypes may be the only way to coordinate behaviour and maintain cooperation. Indeed, both theoretical and experimental [14–17] work have shown that, when deciding whether to cooperate, *intuitive* decision-making is often preferable to careful deliberation.

Whether people use stereotypes when deciding to cooperate, or whether they take the time to learn about others as individuals, depends on a trade-off between ease of decision-making on the one hand and greater benefits from deliberation on the other [3]. For stereotyping to be useful in this context, it must allow people to engage in successful cooperation, while helping them avoid losing out to free-riders and cheats [15]. If stereotypes are too coarse, people risk either cooperating when they should not, or withholding cooperation when it could be productive. If they abandon stereotypes altogether, they lose the ability to engage in intuitive decision-making and generate unnecessary cognitive burdens.

The function of stereotypes, and the dynamics of stereotype formation, have a complex and multifaceted psychological basis that goes beyond cognitive convenience [1–11]. For example, stereotypes also serve a normative function by shaping in-group identity and cohesion [18]. And so, in order to model the evolution of stereotypes, we must account not only for how stereotype attitudes change over time, but how the content of individual and group identities change as well.

The features of identity that determine how people are stereotyped may change as social and political attitudes change, for example social desirability bias can lead to reduced racial polarization [19], exogenous factors such as a shifting media environment can lead to changes in the salience of different aspects of identity (as seen for example in the dynamics of affective polarization [20]), and changes in population structure, such as loss of contact opportunities with out-groups, can lead to induced homophily [21]. At the same time, the groups that individuals identify with may also change over time, for example political affiliation may change to better align with individual preference, and even seemingly fixed aspects of identity, such as religion or ethnicity, can change to better match political or ideological preferences [22,23].

In this paper, we study the evolution of stereotyping as a mechanism for cooperation under cognitive constraints. We consider a form of group reciprocity in which individuals make decisions about whether to cooperate with a partner based on the average observed behaviour of the identity group to which the partner belongs. We explore the evolution of social circles (i.e. the number of people who are not stereotyped, but are instead judged only by their individual behaviour). We also study the evolution of stereotypes themselves (i.e the degree of coarseness or specificity in the stereotypes people employ).

We show that positive stereotypes, in which cooperation with members of a stereotype group is more likely than not, can be maintained if people interact with relatively few (fewer than 100) members of each group. However, we also find that negative judgement bias—in which people tend to be pessimistic about the willingness of members of a stereotype group to cooperate—is common even when stereotypes are positive.

We then show that the coevolution of social circles and stereotype groups undergoes distinct phases, depending on the cognitive costs associated with remembering individual identities, as well as the benefits of cooperation. When cognitive costs are low, social circles are large, and any stereotypes employed tend to be positive. When cognitive costs are intermediate, social circles are smaller, stereotypes are coarser but generally positive, while judgement bias tends to become negative. When cognitive costs are high, social circles shrink and stereotypes become very coarse and negative. Nevertheless, positive stereotypes can be maintained under sufficiently high benefits from cooperation.

By focusing on the content of stereotype attitudes, as captured through the degree of cooperation that emerges between an individual and members of a stereotype group, we are able to connect individual behaviours, such as statistical discrimination [24], to attitudes about groups in the form of stereotypes. And so our model captures both the content of stereotypes and their temporal dynamics as realized through a process of cultural evolution. We end our analysis by exploring the impact of extrinsic shocks on these attitudes. We show that when stereotypes are initially positive, and populations experience a ‘shock’ that reduces the benefits of cooperation, stereotypes can turn negative, resulting in a loss of cooperation and an increase in negative judgement bias, producing attitudinal shifts that can fuel inter-group conflict and mass polarization [8].

## 2. Results

**Figure 1.**
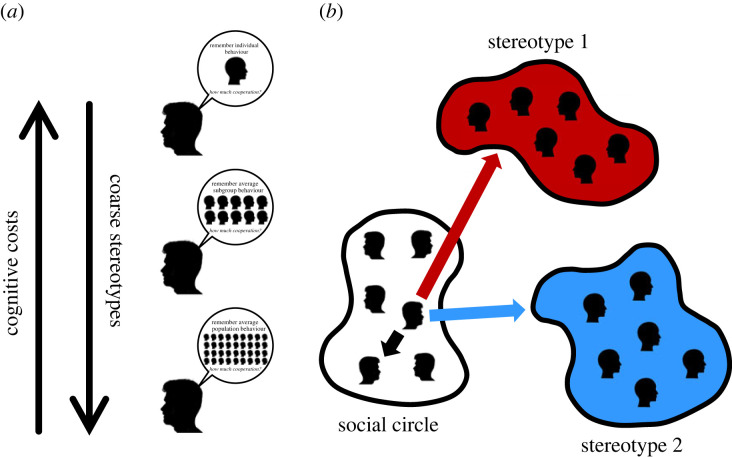
Group reciprocity and stereotyping. (*a*) When a player decides whether to help someone, their decision depends on how much information they have about that person (i.e. on experience from past interactions, the ability to correctly identify other people, the ability to integrate that information to arrive at a decision and so on) [25]. The more information a player has about others, the better they are able to successfully employ reciprocity. In this paper, we distinguish between *direct* reciprocity [26,27], which takes place between members of the same social circle and *group* reciprocity, which takes place with members of stereotyped groups. Under direct reciprocity, players have full knowledge of each others’ identity, and decide whether to cooperate based only on their direct past experience of one another. Under group reciprocity, players decide whether to cooperate based on their experience interacting with all members of the stereotype group. Under direct reciprocity, cognitive costs are higher, but cooperation is easier to sustain, because deviations from cooperation can be dealt with more effectively. Under group reciprocity, cognitive costs are lower, but cooperation is harder to sustain, because deviations from cooperation can only be dealt with in the aggregate. (*b*) We model a population in which *m* individuals belong to one of *G* stereotype groups, and the rest belong to a close social circle of (*N* − *m*) players. When a focal player interacts with a member of a stereotype group (stereotype 1, red background; stereotype 2, blue background), they use the average behaviour of that group to decide whether to cooperate (group reciprocity). When a focal player interacts with a member of their social circle (white background), they use the past behaviour of that individual to decide whether to cooperate (direct reciprocity).

In order to capture the role of stereotyping in social interactions, we assume that people may treat one another differently based on their identity/stereotype group or based on whether they are a part of a close social circle (figure 1). When discussing the model we define the ‘group’ as the set of individuals with the same stereotype who engage in social interactions with a focal individual. The ‘group size’ is, therefore, the number of individuals from a given stereotype group who a focal player interacts with. In reality, the number of people who share a stereotype (but do not interact with a given focal individual) may be much larger than the group size of the model. If two people belong to the same social circle, we assume that they know each other as individuals, and interact based on their direct experience of one another (direct reciprocity). By contrast, when interacting with a partner outside of their social circle, we assume that people make decisions based on stereotypes (i.e. using assumptions about the identity group to which the other person belongs: group reciprocity).

We focus on cooperative social interactions taking place in a game theoretic setting, between a focal player and members of different stereotype groups. We assume that a focal player’s decision to cooperate depends on their strategy, which takes account of the average behaviour of the stereotype group to which their partner belongs. We capture this type of interaction through an iterated pairwise donation game [28–30] played in a population of total size *N*, in which *m* ≤ *N* players are distributed equally among *G* stereotype groups, and the remaining (*N* − *m*) players form the focal player’s close social circle. We assume that the focal player interacts with members of their close social circle using direct reciprocity. By contrast, the focal player interacts with each of *n* = *m*/*G* players in a given stereotype group using group reciprocity. In the extreme case that all players are treated as a member of the same stereotype group, *G* = 1, social interactions with anyone outside of the focal player’s social circle take the form of generalized reciprocity, where willingness to help another is determined by prior receipt of help regardless of the identity of the partner [31].

In order to connect our game theoretic analysis to the wider literature on stereotypes [1–11], we characterize the output of our model in three distinct ways. First we describe the *warmth* of a stereotype as the realized level of cooperation between individuals and members of a stereotype group. Second, we describe the *judgement bias* as the degree of optimism or pessimism about whether a member of a given stereotype group will cooperate. Finally we describe the *coarseness* of a stereotype in terms of the number of people who a focal individual interacts with based on their membership of a given group. We define each of these quantities mathematically below, and show how they coevolve across different environments.

### (a) Rules of the game between groups

Social interactions both within a social circle, and between a focal individual and members of a stereotype group, are assumed to occur via a repeated pairwise donation game [30]. Within a social circle, we make the standard assumption that all pairs of players engage in a repeated game, and use memory-1 strategies to condition their behaviour on past experience, in a way that allows for stable cooperative interactions [30,32–34]. The game dynamics between a focal individual and a stereotype group occur with members of the group being drawn at random, with both players then deciding either to cooperate by paying a cost *C* in order to donate a benefit *B* to their co-player (where *C* < *B*), or else to defect and donate nothing. We assume that the game consists of infinitely many such interactions so that every player in the population gets the opportunity to help (i.e. cooperate with) every other member of the population, and vice versa, resulting in a total payoff for each player in each ‘round’ of the game due given by

$$\begin{array}{rl} & \mathrm{payoff}\;\mathrm{from}\;\mathrm{group}\;\mathrm{reciprocity}\\ & \;=\mathrm{total}\;\mathrm{benefit}\;\mathrm{received}\;\mathrm{from}\;\mathrm{being}\;\mathrm{helped}\;\\ & \;-\;\mathrm{total}\;\mathrm{cost}\;\mathrm{paid}\;\mathrm{due}\;\mathrm{to}\;\mathrm{helping} \end{array}$$

In addition to interactions between members of different stereotype groups, interactions may occur between members of the same social circle through direct reciprocity. And so the total payoff to an individual depends on their payoffs from group reciprocity, as well as their payoffs from direct reciprocity with members of their social circle, and on the cognitive costs of engaging in both types of interaction [35,36] (see below).

We assume that over time, players engage in a very large numbers of interaction ‘rounds’. And so, in our analysis, we treat the system as an infinitely repeated donation game (see Methods). We discuss relaxing this assumption in electronic supplementary material, section S5, and show that our results hold under finitely repeated games. We further assume that players can update their behavioural strategy via imitation of other players [37] (see Methods). We begin by analysing the evolutionary dynamics of cooperation in the presence of fixed stereotype groups and in the absence of social circles (*m* = *N*). We then expand our analysis to consider the evolution, over longer time scales, of social circles, and finally the co-optimization of social circles and the number of stereotype groups present in the population.

### (b) Stereotyping

In order to study the evolution of stereotyping, we model two kinds of social interaction. First we model interactions between members of stereotype groups of size *n*, which we assume occur via *group reciprocity*. Second, we also model interactions between members of the same social circle, which we assume occur in general via *direct reciprocity*. We begin by studying the evolution of group reciprocity between members of fixed stereotype groups (see Methods). We then study the evolution of stereotype groupings and social circles. Initially, we assume that interactions between members of the same social circle are always cooperative. We relax this assumption in the electronic supplementary material and show that, when cooperation between members of the same circle produce lower benefits, our results are qualitatively unchanged (electronic supplementary material, section S3.7).

When interacting with others according to their stereotype, a focal player makes a decision to cooperate based only on their experience of that group’s *average* behaviour. We identify the propensity of a focal individual to cooperate with a member of a group according to their stereotype of that group. Although we initially assume that this propensity is based on the experience of the focal player, we also explore scenarios in which it is derived from the average experience of *all* members of the population—which leads to a decline in the warmth of stereotypes (see electronic supplementary material, section S4).

We assume that players make their decision about whether to help a given member of a given group by adopting one of a broad family of behavioural strategies, which cooperate with a probability that depends linearly on the average amount of help the player has received from members of that stereotype group in the preceding round:

2.1 $$p_{k}^{i}=s\frac{k}{n}+r.$$

Here $p_{k}^{i}$ is the probability that player *i* helps a member of a given stereotype group of *n* individuals, of which *k* cooperated in the preceding round. The parameter *r* determines the baseline rate of cooperation (i.e. the probability of cooperating even when no member of the group helped in the previous round) and *s* determines the rate of change of cooperation with help received (i.e. the marginal increase in the probability of cooperation with each additional player who cooperated in the preceding round), where 0 ≤ *r* ≤ 1 and −*r* ≤ *s* ≤ 1 − *r*. In the first round we assume that players help with a probability given by their ‘baseline’ rate of cooperation *r* as given in equation (2.1), however, because we are considering an infinitely repeated game with noise our analytical results are insensitive to this assumption (see Methods).

The family of conditional strategies, equation (2.1), reduces to the classic pairwise tit-for-tat strategy when *n* = 1, *r* = 0 and *s* = 1, to always cooperate when *s* = 0 and *r* = 1 and to always defect when *s* = *r* = 0. When *n* > 1 along with *s* = 1 and *r* = 0, a group-level strategy analogous to tit-for-tat arises, under which both mutual cooperation and mutual defection are stable when the strategy is adopted by all players, with stochastic switching between the two in the presence of noise [33]. It also includes generous strategies [30,38–40] as well as extortionate strategies [28,41,42]. More generally, when *s* = 0, equation (2.1) reduces to an unconditional strategy in which individuals cooperate with fixed probability. Note that if we restrict ourselves to unconditional strategies, cooperation cannot evolve in this system, absent some additional cooperation promoting mechanism such as indirect reciprocity or kin selection [26]. And so our choice of equation (2.1) represents the simplest family of strategies that can produce cooperation through group reciprocity, without the requirement for additional assumptions.

We assume that all players interact with the same number of players from a given stereotype group, *n* = *m*/*G*. We also assume that stereotyping is reciprocal, meaning that if player *i* treats player *j* as a stereotype, then player *j* also treats player *i* as a stereotype (though these players may stereotype one another in different ways). Equation (2.1) describes a strategy for engaging in *group reciprocity* between stereotype groups. We study the evolutionary dynamics of group reciprocity between a large number of such groups, with particular focus on the average rate of cooperation among groups.

Stable cooperation requires all members of all stereotype groups to adopt a strategy *s* = 1 − *r*, which simply means that a player will cooperate with certainty if everyone in the partner’s stereotype group cooperated in the previous round. If such a strategy is used by all players then, when *k* = *n* ( meaning that all players cooperated in the preceding round) every member of each group will help every member of each other group in the next round. And so, everyone will continuously cooperate. A group in which all players use a strategy with *s* = 1 − *r* is, therefore, said to be *cooperative*.

Conversely, stable defection requires all players adopt a strategy *r* = 0. This means that when *k* = 0, no player will help any other player, and everyone will defect. A group in which all players use a strategy with *r* = 0 is, therefore, said to be *non-cooperative*.

### (c) Evolution of group reciprocity

The evolutionary dynamics among stereotype groups occurs via a process of imitation and random innovation. Players copy one another’s strategy (equation (2.1)) with a probability that depends on the average payoff each player received from interactions with all members of the population in the infinitely repeated game described above. We assume that, when players update their strategy, they imitate individuals from other stereotype groups at rate *α*, and otherwise imitate individuals who belong to their own stereotype group (see Methods). As a result, the probability of imitating a member of their own stereotype group is (*n* − 1)/((*n* − 1) + *αG*). Throughout we assume imitation of other stereotype groups occurs at rate *α* = 0.5/*N*. We explore the effects of varying *α* in electronic supplementary material, figure S4.

Under this process, the strategy space described by equation (2.1) allows for only fully cooperative, or fully non-cooperative Nash equilibria, which means only these behaviours can resist invasion [33] (see Methods). Because the only available Nash equilibria are weak, the system contains no strict evolutionary stable strategies, and over long time scales the system cycles between cooperation and defection (see electronic supplementary material, section S1) [33]. Cooperative strategies, for which *s* = 1 − *r*, can resist invasion provided *s* > 1 − *ρ* where *ρ* describes the robustness of cooperation and is approximated by *ρ* ≈ 1/2(*Gα*/*n*^2^)((*B*/*C*) − 1) when *n* ≫ 1 (see Methods, where we also provide the full analytical form of *ρ*). In other words, the robustness of cooperative strategies declines rapidly with stereotype group size *n*, but increases with the rate of out-group imitation, *α*, and the ratio of benefits to costs of cooperation, *B*/*C*.

**Figure 2.**
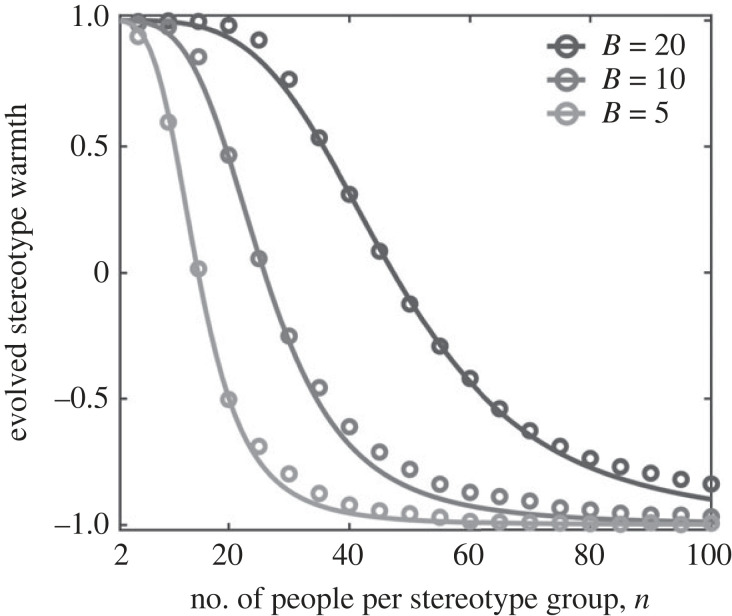
Positive and negative stereotypes. As the number of players per-stereotype group, *n*, increases, the robustness of cooperation, *ρ*, and the resulting stereotype warmth, *W*_*g*_ decline. We approximated the average stereotype warmth *W*_*g*_ that will arise in a population over the course of evolution, using equation (2.3) (solid lines) for different levels of benefit from cooperation *B*, keeping the cost of cooperation fixed, *C* = 1. As the ratio *B*/*C* increases, the number of people per stereotype group with whom a player interacts which can sustain a positive stereotype increases. When *B*/*C* = 20, positive stereotypes for groups of up to approximately 50 individuals can be sustained. When *B*/*C* = 5, positives stereotypes for groups of only approximately 10 individuals can be sustained. The analytical approximation is compared to individual based simulations (dots) for stereotype groups of between 2 and 100 individuals. Results shown are for *G* = 10 groups, with selection strength *σ* = 10 and out-group imitation rate *α*/*N* = 0.5. We ran Monte Carlo simulations with evolution describe via a Moran process with fitness based on group identity as described in the main text. Total population size was *N* = *nG* and global mutations occurring at rate *μ* = 1/*N*. Simulations ran for 10^4^ generations with expected cooperation rate calculated from 10^4^ sample paths. The structural constant *β* was calculated numerically and has values *β* = 0.00032 when *B* = 20, *β* = 0.00073 when *B* = 10 and *β* = 0.0011 when *B* = 5. The constant *β* was calculated numerically based on the value of *n* that produced *ρ* = 0.5 given *B* and *C*.

Similarly, non-cooperative strategies, for which *r* = 0, are stable provided *s* < 1 − *ρ*. No other type of strategy can resist invasion (see Methods), and so the long-term evolutionary dynamics involve repeated shifts between cooperation and defection, at a rate that depends on *ρ* [33]. Under such dynamics the long-term average rate of cooperation can be approximated by Stewart & Plotkin [33],

2.2 $$\Pi_{c}\approx \frac{\rho^{2}}{\rho^{2}+\beta {\left(1-\rho \right)}^{2}},$$

(figure 2) where *β* is a structural constant that depends on the strength of selection, *σ*, and can be estimated numerically [33] (see Methods). We first use this approximation to study how the warmth of stereotypes change as a function of *n*, the number of people from a given stereotype group who a player interacts with. We then apply those results to study the evolution of social circle size, (*N* − *m*), and the number of stereotype groups *G*.

### (d) Stereotype warmth and judgement bias

We characterize stereotypes according to their warmth (i.e. whether the stereotype is positive or negative about the group being considered) and by their judgement bias (i.e. the degree of optimism or pessimism about the group given their past actions) [43]. Both stereotype warmth and judgement bias are characterized in terms of the amount of cooperation between a focal player and members of a stereotype group. We define a stereotype to have a positive warmth if a player is more likely to cooperate with a member of a stereotype group than not. Specifically, we write the stereotype warmth as $W_{g}^{i}=2\Pi_{c}^{i}-1$ where $\Pi_{c}^{i}$ is the average rate of cooperation between a focal player *i* and members of the group *g*.

Over long time scales, the average stereotype warmth for the population that arises from the evolutionary dynamics described above can be approximated as *W*_*g*_ ≈ (*ρ*^2^ − *β*(1 − *ρ*)^2^)/(*ρ*^2^ + *β*(1 − *ρ*)^2^) (see electronic supplementary material, section S1).

Figure 2 shows how stereotype warmth changes with the size of the stereotype group *n*. We see that even when the benefits of cooperation are large (*B*/*C* = 20), stereotypes become negative when players interact with more than approximately 50 members of a given stereotype group. This is because group reciprocity becomes harder to maintain as stereotypes become coarser—i.e. if a stereotype group is large, the presence of a single defector reduces the tendency of outsiders to cooperate with a large number of people—and so lower levels of cooperation evolve at equilibrium.

In addition to stereotype warmth, we also explore the degree of judgement bias encoded in the behavioural strategies that evolve among players engaging in group reciprocity. We define a strategy (i.e an attitude towards a particular stereotype) to have a positive judgement bias if the evolved strategy is ‘optimistic’ about the behaviour of members of the stereotype group. In this context we call a strategy optimistic if, for a given level of cooperation from the group *k*/*n*, the focal player is more likely to cooperate than they are to be cooperated with. In terms of iterated game strategies, a player who uses *s* = 1 and *r* = 0—which can be understood as a multi-player generalization of tit-for-tat [38]—is neutral with respect to judgement bias since it cooperates in response to cooperation and defects in response to defection. A grim trigger strategy has negative judgement bias, since it always defects in response to a single instance of defection [44]. A generous strategy has positive judgement bias, since it tends to cooperate even in response to defection [30,45].

We define the judgement bias of a focal player *i* interacting with members of a group *g* as $J_{g}^{i}=(4/(n+1))\sum_{k=0}^{n}(p_{k}^{i}-k/n)$, which in turn depends on the baseline rate of cooperation *r*_*i*_ and the slope *s*_*i*_ of the player’s strategy (equation (2.1)). We show that the average judgement bias for the population that arises from the evolutionary dynamics at equilibrium can be approximated as *J*_*g*_ ≈ *W*_*g*_*ρ* + (*W*_*g*_ − 1)/2 (see electronic supplementary material, section S2).

**Figure 3.**
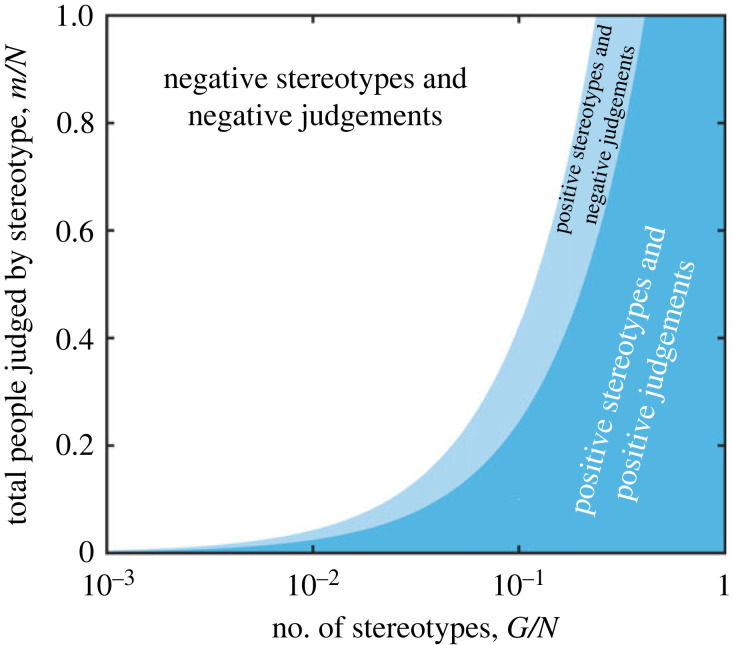
Stereotyping and judgement bias. The evolution of positive stereotypes, *W*_*g*_ > 0, and positive judgement bias, *J*_*g*_ > 0 depends on the number of individuals per stereotype group, *n*. This in turn depends on the proportion of the population who are stereotyped *m*/*N* and on the number of stereotype groups *G*/*N*. Positive stereotype warmth (blue regions) is easier to produce than positive judgement bias (dark blue region). Both are easier to evolve when the number of stereotype groups is large enough that the number of stereotyped people that a player interacts with per group is small (i.e. when the ratio *n* = *m*/*G* is sufficiently small). This suggests when stereotyping is common, and stereotypes are coarse, attitudes towards stereotyped individuals will tend to be negative. Plots shown are based on equilibrium cooperation rates (see Methods) with *B* = 5 and *C* = 1.

Key to understanding this evolution is the trade-off between the efficacy of group reciprocity on the one hand (i.e. how much cooperation can be maintained among a given set of stereotype groups, as described in figures 2 and 3) and the cognitive costs associated with different kinds of behavioural strategies on the other (see figure 1).

Figure 3 shows the conditions under which positive judgement bias and positive stereotype warmth can evolve. We see that positive stereotype warmth is easier to achieve than positive judgement bias—that is, behavioural strategies that are ‘optimistic’ about people are the hardest to evolve. Both positive judgement bias and stereotype warmth are easiest to evolve when the number of stereotypes, *G*, is large and the number of people being stereotyped, *m*, is small. This reflects the fact that it is only when players interact with relatively small numbers of people per stereotype group, *n* = *m*/*G*, that cooperation can be maintained (figure 2).

### (e) Cognitive capacity

So far we have considered the evolution of group reciprocity and stereotypes for fixed social circle size *N* − *m* and a fixed number of stereotype groups *G*. This assumption may be valid over time scales of a few generations, in which social attitudes may shift while the population structure remains fixed. However, over longer time scales, we must also ask how social circles and stereotype group structures themselves change.

In particular, we assume that players remember the identity and past behaviour of members of their social circle, while they only remember the group identity and group average behaviour of those they stereotype. The latter represents a lower cognitive cost than the former. To quantify this, we calculate the information required to store the identity of each member of a social circle of size *N* − *m*, along with the group identities of *m* stereotyped individuals distributed across *G* groups:

2.3 $$\begin{array}{rl} & I_{s}(m,G)=\log_{2}\left[A\frac{G}{N}\right]+\frac{G}{N}\log_{2}\left[\frac{m}{G}+1\right]\\ \mathrm{and}\; & I_{c}(m)=\log_{2}\left[A\frac{N-m}{N}\right]+\frac{N-m}{N} \end{array}\}$$

where *I*_*s*_ is the information per population member required to store a player’s group reciprocity strategy, *I*_*c*_ is the information per population member required to store a player’s strategy for interacting with their social circle. The constant *A* scales the information required to store the identity of a given individual (see electronic supplementary material, section S3.1).

## 3. Evolution of social circles

In order to study the evolution of social circles we assume that we can separate the time scale of behavioural strategy evolution from the time scale of social circle evolution. In particular, we assume that behavioural strategies quickly reach an equilibrium described by equation (2.2) (see electronic supplementary material, section S3). We then model the evolution of social circles, i.e. the proportion of players *m*/*N* who are stereotyped, using the framework of adaptive dynamics.

Under this framework the fitness of a mutant individual *i*, who stereotypes *m*_*i*_ individuals is given by

3.1 $$\begin{array}{rl} w_{i} & =\left(1-\frac{m_{i}}{N}\right)(B-C){\left(1-C_{m}\right)}^{I_{c}(m_{i})}\\ & \;+\frac{m_{i}}{N}(B-C){\left(1-C_{m}\right)}^{I_{s}(m_{i},G)}\Pi_{c}(n,G) \end{array}$$

where *C*_*m*_ scales the cognitive cost of storing strategy and identity information about individuals and their stereotype groups, and $\Pi_{c}$ is the average rate of cooperation among stereotype groups, due to the resident strategy for the population as given in equation (2.2). We have assumed that players always cooperate with members of their social circle, although we relax this assumption in electronic supplementary material, section S3.7.

In the adaptive dynamics limit *N* → ∞ the proportion $m_{i}/N=x_{m}^{i}$ is a continuous variable and we can study the evolutionary dynamics of social circles by evaluating the selection gradient $\partial w_{i}/\partial x_{m}^{i}{|}_{x_{m}^{i}=x_{m}}=0$ where *x*_*m*_ is the resident value for the population.

**Figure 4.**
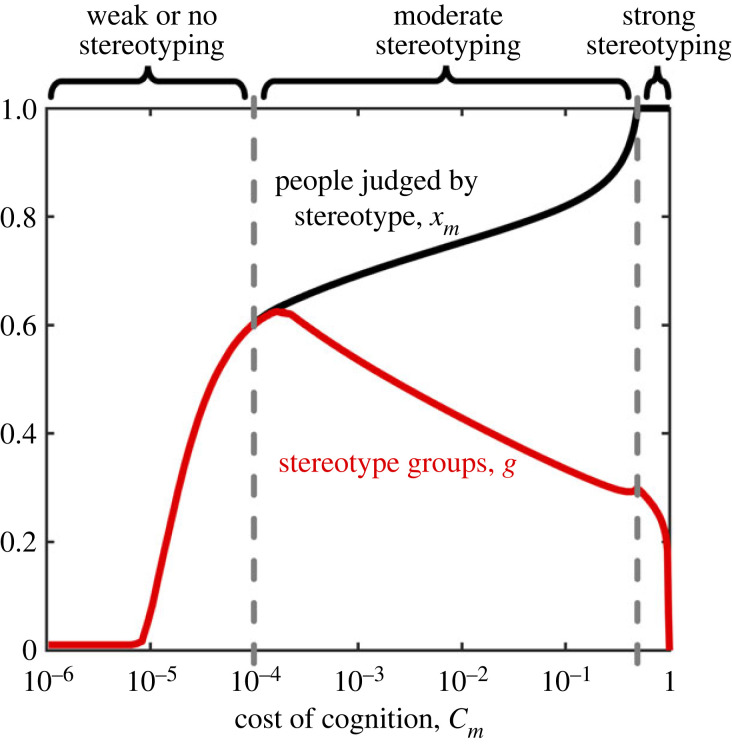
Evolutionary optimal stereotype groups and social circles. Stereotyping takes different forms depending on the cost of cognition *C*_*m*_ and the benefit cost ratio of cooperation, *B*/*C*. We calculated the evolutionary stable social circle size, 1 − *x*_*m*_ from equation (3.1) (see Methods) as a function of the number of stereotypes per capita, *g*, where we have set *x*_*m*_ = *m*/*N* and *g* = *G*/*N*, and taken the limit *N* → ∞ (see electronic supplementary material, section S3). We then calculated the value of *g* that maximizes fitness, to give the evolutionary optimal social circle size and number of stereotype groups for a given set of parameters. Evolutionary optimal social circle size (black line) and number of stereotype groups (red line) as a function of cognitive costs *C*_*m*_. When cognitive costs are small (here *C*_*m*_ < 10^−4^), there is one stereotype group per stereotyped individual, indicating weak or no stereotyping. For intermediate cognitive costs (here 0.0001 < *C*_*m*_ < 0.5) optimal stereotypes become increasingly coarse (smaller values of *g*) and social circles shrink (higher values of *x*_*m*_). For high cognitive costs (here *C*_*m*_ > 0.5) social circles vanish (*x*_*m*_ = 1) and everyone is judged via coarse stereotypes. Evolutionary optima are calculated numerically (see Methods) with *B* = 5 and *C* = 1, *α* = 0.5 and *β* = 0.0011.

In the supplement we show that, for a fixed number of stereotype groups per person *G*/*N* = *g*, there is a single equilibrium social circle size (electronic supplementary material, section S3), with social circles tending to be smaller when cognitive costs are higher. However, we also find that there is an *optimum* number of stereotype groups which maximizes population fitness (electronic supplementary material, section S3). In figure 4, we study how the equilibrium social circle size, and the optimum number of stereotype groups, co-vary as a function of cognitive costs. The equilibrium social circle size captures the proportion of people treated as individuals rather than stereotyped, while the optimum number of stereotype groups captures the coarseness of the stereotypes applied to those outside of the social circle. And so we can understand a *decrease* in the optimum number of stereotype groups as different facets of group identity being increasingly ‘lumped together’. In the most extreme form of stereotyping there is only a single stereotype group, and all people are treated as either a member of the social circle, or as simply ‘other’.

We find that the nature of stereotyping changes qualitatively as the cognitive costs of behavioural strategies increase. There is a threshold value of *C*_*m*_ below which *x*_*m*_ = *g* (i.e. where it is optimal to have ‘stereotype groups’ composed of only a single individual). In this case there is really no stereotyping, because players in effect engage in direct reciprocity with ‘stereotyped’ individuals. Above this threshold, there is a range of values for *C*_*m*_ such that *x*_*m*_/*g* > 1 and *x*_*m*_ < 1 (i.e. genuine stereotyping occurs, while social circles are also maintained). As *C*_*m*_ increases, social circles get smaller and stereotype groups decrease in number (i.e. groups contain more people and so stereotypes become increasingly coarse). Finally there is a value of *C*_*m*_ above which social circles vanish (i.e. *x*_*m*_ = 1), and the number of stereotype groups rapidly declines, so that any cooperative interactions resemble generalized reciprocity [31].

Notably we find that increasing the relative benefits of cooperation with stereotypes, *B*/*C*, has similar effects on stereotyping to increasing *C*_*m*_ (electronic supplementary material, figure S6). This occurs because, as the benefits of cooperation increase, it is easier to maintain high levels of cooperation via group reciprocity and so, for fixed cognitive costs, it is preferable to stereotype more individuals, more coarsely.

### (a) Environmental shocks

**Figure 5.**
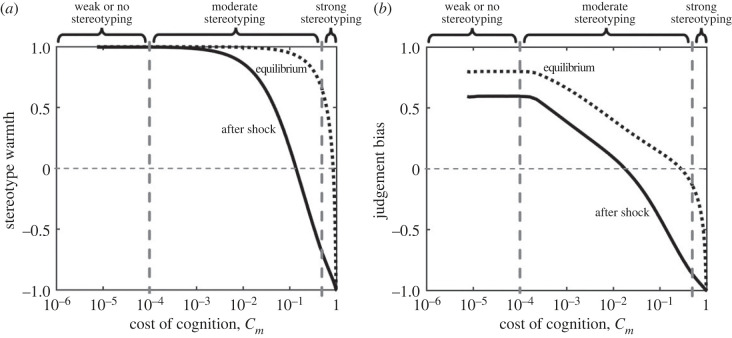
Stereotypes after environmental shocks. We explored what happens to stereotype warmth and judgement bias before and after an extrinsic shock, consisting of a reduction in the cost-benefit ratio from *B*/*C* = 5 to *B*/*C* = 2.5. We assume that social circle size, 1 − *x*_*m*_, and number of stereotype groups per person, *g*, remains fixed at the evolutionary optimum for the population before the shock. We then calculate the equilibrium cooperation rate (equation (2.2)) for the system before and after the shock as a function of the cost of cognition *C*_*m*_. (*a*) We see that stereotype warmth *W*_*g*_ is positive before the shock (dashed line) unless the cost of cognition is very high (*C*_*m*_ ∼ 1). However, after the shock, stereotypes become more negative (solid line), and for intermediate values of *C*_*m*_, stereotype warmth switches from being positive to negative after the shock. (*b*) By contrast judgement bias *J*_*g*_ becomes negative at equilibrium for low values of *C*_*m*_ (dashed line), and becomes negative after a shock (solid line) for intermediate values of *C*_*m*_. Evolutionary optima before and after the shock are calculated numerically with *α* = 0.5 and *β* = 0.0011 (before shock) and *β* = 0.0038 (after shock).

Finally we consider how stereotypes change in response to an extrinsic shock, in which the benefits of cooperation are reduced compared to historical values. We assume that over a long time scale, a population reaches an optimum level of stereotyping given the costs and benefits of cooperation and the costs of cognition. We then analyse how stereotype warmth and judgement bias shift when the benefits of cooperation are reduced, while keeping social circle size and number of stereotype groups fixed at their previous values (figure 5). Note that the stereotypes that emerge in response to such a shock in general differ from the stereotypes that evolve when social circles and stereotype groupings are allowed to change.

We find that such shocks tend to produce a negative shift in both stereotype warmth and judgement bias and, most importantly, can result in positive stereotypes becoming negative. The extent of this effect depends on the pre-shock equilibrium and is most pronounced when there is a mixture of coarse stereotyping and large social circles (figure 5*c*).

## 4. Discussion

Stereotyping is a common feature of human decision-making and is often seen as having negative social consequences [1–11]. However, stereotyping can produce benefits by reducing the cognitive load of decision-making, aiding coordination or signalling trust [3,14–17]. Here, we show that stereotyping can evolve via a process of cultural evolution as a mechanism to enable cooperation while minimizing the cognitive costs of recalling the identities and past actions of large numbers of individuals. We show that, unless cognitive costs are very large (figure 3), cultural evolution is effective at producing *positive* stereotypes, which maintain cooperation among individuals who stereotype one another. However, we also find the positive stereotypes can quickly turn negative after environmental ‘shocks’ i.e. following a reduction in the benefit-cost ratio of cooperative interactions (figure 4).

This phenomenon, in which increased adversity leads to a loss of cooperation with (and increasingly negative attitudes towards) out-groups, is consistent with empirical and theoretical accounts of inter-group conflict [9,46,47] and a growing body of work focused on the global trend towards mass political polarization [20,48–52]. What our results highlight is that such an increase in negative attitudes towards out-groups can arise due to the dynamics of cultural evolution, when there is a mismatch between the optimal state of the system before and after an exogenous shock. Such an effect cannot be captured by a ‘static’ model of behaviour, since it arises even when a withdrawal of cooperation due to adverse conditions is not rational. The mismatch which evolves may be self-correcting over long time scales, if the population is able to evolve to a new cooperative optimum, in which case negative stereotyping may be transient. However, in practice there may be significant inertia preventing such optimization, when it requires widespread changes in behaviour or the way shared stereotype groups are defined, for example.

Our results can be viewed in contrast to previous models of cooperation in the presence of tag-based strategies [53] or green beard effects [54]. Under such models information about identity can facilitate cooperation by providing an indicator of similarity, and identity itself evolves alongside cooperative behaviour. By contrast under our model information about identity is given exogenously, and stereotypes evolve due to a trade-off between the cognitive costs and the cooperative benefits of keeping track of that identity. Future work may look to bridge the gap between these two perspectives, with individual identity modelled as comprising both fixed and evolving features [52].

We interpret stereotypes through the lens of warmth—determined by how likely an individual is to cooperate with a member of a given stereotype group—and through judgement bias—the degree of optimism or pessimism about the likelihood of others to cooperate based on their stereotype. Under this model, stereotype warmth reflects the realized behaviour of an individual towards members of a stereotype group, while judgement bias reflects the underlying behavioural strategy of an individual when interacting with members of a stereotype group, (equation (2.1)). We do not attempt to model the individual characteristics (e.g. race, religion, language) that determine membership of a given group, although we implicitly assume that such variation determines group membership. While willingness to cooperate and judgement bias are not identical to stereotype content, we assume that they are translated into stereotype content over time (e.g. groups that compete for resources are less likely to cooperate and so feel less warmth towards one another [4,5]). In this context, it is notable that negative judgement bias tends to emerge before negative stereotype warmth (figures 3 and 5) meaning that high levels of cooperation can be maintained with members of a stereotype group, even when attitudes towards the group are pessimistic. And so if judgement bias drives broad negative characterizations of members of a stereotype group, this may initially occur without loss of cooperation with members of that group.

Our work focuses on the interaction between group reciprocity and stereotypes. However, a key feature of stereotyping is that it involves shared assumptions about members of a group that are disconnected from personal experience (see electronic supplementary material, section S4). In the context of our model, such shared assumptions determine which stereotype group an individual is assigned to. However, we have not attempted to model baseline variation in this form of stereotype content. In particular, variation in perceived competence [4,5] has been shown, along with stereotype warmth, to predict stereotype content across cultural contexts [2]. While we explicitly identify the degree of cooperation with the warmth of a stereotype, we do not model variation in competence across groups. Form the perspective of our model, competence constrains the baseline willingness of individuals to engage in cooperation with different groups. And so, our results can be seen to be complementary to social psychological accounts of stereotyping. We address the evolutionary question under a simplified scenario—when there is no variation in competence between groups, how much warmth/cooperation will evolve? Future work will naturally look to the effect of variation in competence on the evolution of group reciprocity and stereotypes.

Our model focuses on the evolution of cooperation between groups as a proxy for inter-group attitudes. In particular, we model cooperation via group reciprocity arising from repeated interactions between an individual and members of an out-group, which is appropriate for modelling (e.g. how stereotypes evolve among human communities living side by side). An obvious alternative game theoretic modelling framework to capture inter-group dynamics is offered by indirect reciprocity, which makes use of highly stylized reputation norms to reach conclusions about the outcome of one-off interactions among players with access to a very limited set of behavioural strategies. Recent work has begun to integrate the mathematical frameworks of direct and indirect reciprocity [55], and extending this approach to incorporate stereotyping will be a productive direction for future work. Our model also has implications for phenomena such as statistical discrimination [24] and future work will look at the dynamics of the behaviours that result from stereotypes from an empirical point of view. We will also look to extend the model to incorporate the role of stereotypes in promoting group cohesion [18], particularly in the context of economic shocks, where stronger norm enforcement [56,57] coupled with declining cooperation with stereotype groups can mutually reinforce each other.

Understanding the cause and consequence of people viewing one another as stereotypes is increasingly important, as geography ceases to limit close social interaction, different forms of identity become salient, and diverse political and social movements come into conflict. The lens of imitation dynamics and cultural evolution allows us to explore how interventions seeking to reduce inter-group conflict and negative stereotyping are likely to play out over both short and long time scales. In particular, we show that, when a population is easily able to reach an evolutionary optimal state, stereotypes will often have positive warmth, and maintain high levels of cooperation. However, if stereotype groups are inflexible, this cooperation may easily be lost in response to extrinsic shocks. And so, to prevent the negative consequences of stereotyping, it may not be necessary to discourage stereotyping altogether, but rather to encourage adaptability in the way people stereotype each other.

## 5. Methods

Here, we provide analytical results on the evolutionary robustness of cooperative and non-cooperative strategies under our model of stereotypes. Further details of simulations and the adaptive dynamics analysis can be found in the electronic supplementary material.

### (a) Payoffs in the infinitely iterated donation game

We first consider the dynamics of repeated interactions under fixed strategies (i.e without evolution). We assume that players engage in an infinitely iterated, asynchronous pairwise donation game with members of a stereotype group. The first player in a given interaction chooses whether to pay a cost *C* and donate a benefit *B* to the second player in the interaction. We assume that this game occurs in a population such that every player has the opportunity both to donate help and to receive help from every other member of each stereotype group equally (i.e all possible pairwise interactions occur with the same probability).

We consider a focal player *i* who divides their partners into stereotype groups, and uses a strategy $p_{k}^{i}$ to decide whether to donate to any given member of that stereotype group. Her strategy takes account of the total number of players *k* in the group who cooperated with her across the preceding *n* = *m*/*G* interactions as described by equation (2.1). In any given round of interactions with the *n* = *m*/*G* members of a stereotype group, player *i* can choose to donate between 0 and *mC*/*G* to the group, and similarly members of the group (from the focal player’s perspective) choose to donate between 0 and *mB*/*G* to the focal player.

And so player *i* can treat their interactions with a partner from a given stereotype group as a two-player, infinitely iterated, *n* + 1 choice game of the type studied in [34] and elsewhere. If we write $v_{lk}^{t}$ for the probability that, in round *t*, player *i* donated *l* times to members of the stereotype group and members of the stereotype group donated to the player *k* times then the time evolution of plays in the multi-choice game is described by

5.1 $$v_{lk}^{t+1}=\sum_{\;j_{p}}\sum_{\;j_{g}}p_{\;j_{p}j_{g}}^{l}q_{\;j_{g}j_{p}}^{k}v_{\;j_{p}j_{g}}^{t},$$

where $p_{\;j_{p}j_{g}}^{l}$ is the probability of player *i* making *l* donations given that they made *j*_*p*_ donations and members of the stereotype group made *j*_*g*_ donations in the preceding round, while $q_{\;j_{g}j_{p}}^{k}$ is the probability that members of the stereotype group made *k* donations to player *i* under the same conditions. Note that $q_{\;j_{g}j_{p}}^{k}$ in general depends on the strategies of *m*/*G* different individuals and is not itself a strategy, but the effective strategy of the sub-group from the perspective of *i*. However, because it is the probability of an event if we sum over all possible events (i.e. all possible donations from the stereotype group to *i*) we must have $\sum_{k=0}^{n}p_{\;j_{g}j_{p}}^{k}=1$ so that

5.2 $$\sum_{k}v_{lk}^{t+1}=\sum_{\;j_{g}}\sum_{\;j_{p}}p_{\;j_{p}j_{g}}^{l}v_{\;j_{p}j_{g}}^{t}.$$

If we now assume a strategy $p_{k}^{i}=r+s(k/n)$ independently determines each decision to contribute (or not) on the part of *i* over all of their *n* interactions with the stereotype group then

5.3 $$p_{\;j_{g}j_{p}}^{l}=\left(\begin{array}{c} n\\ l \end{array}\right){\left(r+s\frac{\;j_{g}}{n}\right)}^{l}{\left(1-r-s\frac{\;j_{g}}{n}\right)}^{n-l},$$

if we use equation (5.2) in equation (5.3), multiply both sides by *l* and sum over *l*/*n* we recover

5.4 $${\left\langle j_{p}\right\rangle }_{t+1}=rn+s{\left\langle j_{g}\right\rangle }_{t},$$

where 〈*j*_*p*_ 〉 _*t*_ is the expected number of times the focal player contributes in round *t* and 〈*j*_*g*_ 〉 _*t*+1_ is the expected number of times the group contributes. If we assume a small amount of noise in the execution of play so that the Markov chain describing the sequence of plays has a unique stationary distribution (i.e does not contain multiple absorbing states), then in an infinitely iterated game at equilibrium we have [42]

5.5 $$\langle j_{p}\rangle =rn+s\langle j_{g}\rangle .$$

The expected number of donations received by *i* at round *t* from a given member of a stereotype group is 〈*j*_*g*_ 〉 _*t*_ and the expected number of donations made is 〈*j*_*g*_ 〉 _*t*_. Thus the expected average payoff to player *i* once the game has reached equilibrium such that equation (5.6) holds is

5.6 $$\pi_{i}=B\langle j_{g}\rangle -C\langle j_{p}\rangle .$$

### (b) Payoff to an invader

We now consider a resident strategy invading in a population comprising *G* stereotype groups of fixed size *n*, in which all interactions between members of different groups occur via group reciprocity. In particular, we consider a resident strategy

5.7 $$p_{k}^{r}=s_{r}\frac{k}{n}+r_{r},$$

being invaded by a mutant

5.8 $$p_{k}^{m}=s_{m}\frac{k}{n}+r_{m}.$$

We assume that the resident strategy is used across all stereotype groups, and ask whether the mutant can spread within the population. Under this assumption the behaviour of the resident strategy within a focal stereotype group is described by

5.9 $$\langle j_{r}\rangle =r_{r}n+s_{r}\langle j_{g}\rangle ,$$

whereas the behaviour of the mutant strategy is described by

5.10 $$\langle j_{m}\rangle =r_{m}n+s_{m}\langle j_{g}\rangle .$$

Finally the behaviour of other stereotype groups when interacting with a player withing the focal steretype group is described by

5.11 $$\langle j_{g}\rangle =r_{r}n+s_{r}\frac{n-1}{n}\langle j_{r}\rangle +s_{r}\frac{1}{n}\langle j_{m}\rangle ,$$

Solving equations (5.9)–(5.11) we recover

5.12 $$\begin{array}{rl} & \langle j_{g}\rangle =\frac{\left(1+s_{r})nr_{r}+s_{r}(r_{m}-r_{r}\right)}{s_{r}(s_{r}-s_{m})+n-s_{r}^{2}n},\\ & \langle j_{r}\rangle =\frac{\left(1+s_{r})nr_{r}+s_{r}(s_{r}r_{m}-s_{m}r_{r}\right)}{s_{r}(s_{r}-s_{m})+n-s_{r}^{2}n}\\ \mathrm{and}\; & \langle j_{m}\rangle =\frac{\left(1+s_{r})n(s_{m}r_{r}+r_{m}-s_{r}r_{m})+s_{r}(s_{r}r_{m}-s_{m}r_{r}\right)}{s_{r}(s_{r}-s_{m})+n-s_{r}^{2}n} \end{array}\}$$

The payoff received by the mutant is

5.13 $$\pi_{m}=B\langle j_{g}\rangle -C\langle j_{m}\rangle ,$$

whereas the payoff received by the resident strategy within the focal stereotype group is

5.14 $$\pi_{r}=B\langle j_{g}\rangle -C\langle j_{r}\rangle .$$

Finally the payoff to the resident strategy due to interactions among members of other stereotype groups when interacting with the focal stereotype groups is

5.15 $$\pi_{r}^{*}=B\frac{1}{n}\langle j_{m}\rangle +\frac{n-1}{n}\langle j_{r}\rangle -C\langle j_{r}\rangle ,$$

and the payoff for the resident strategy when interacting with other stereotype groups is

5.16 $$\pi_{r}^{†}=(B-C)\frac{r_{r}}{1-s_{r}}.$$

### (c) Imitation dynamics

We assume that cultural evolution occurs through players imitating other strategies based on payoff [37]. When a mutant is rare the observed payoff of the resident strategy among stereotype groups is approximated by

5.17 $$\phi_{r}=\frac{n-1}{n-1+\alpha G}\pi_{r}+\frac{\alpha G}{n-1+\alpha G}\pi_{r}^{†}.$$

Where the first term describes observation of *n* − 1 other members of their own stereotype group (i.e.we assume people from the same stereotype group form the basis of in-group social learning of group reciprocity) and the second term describes observation of members of other groups. In contrast the observed payoff for the mutant’s own strategy is

5.18 $$\phi_{m}=\pi_{m}.$$

Under the assumed imitation dynamics a player will imitate a mutant in their own group with probability

5.19 $$f_{r\to m}=\frac{1}{1+\exp[\sigma (\phi_{r}-\phi_{m})]},$$

and the condition for invasion is *ϕ*_*m*_ > *ϕ*_*r*_.

### (d) Evolutionary robust strategies

It is possible to show that only a cooperative strategy, for which *r*_*r*_ + *s*_*r*_ = 1 or a non-cooperative strategy, for which *r*_*r*_ = 0, can resist invasion. In order to see this, we first calculate *ϕ*_*r*_ − *ϕ*_*m*_ for an arbitrary resident strategy and non-cooperative invader, *r*_*m*_ = 0. Substituting from equation (5.12) we then find

5.20 $$\begin{array}{rl} \phi_{r}-\phi_{m} & =r_{r}\\ & \;\times \frac{\left(1-s_{m})((1-\gamma )s_{r}(B-Cs_{r})-C(1-s_{r}^{2})n\right)}{\left(1-s_{r})(n-s_{r}(s_{m}+s_{r}(n-1))\right)}. \end{array}$$

Where we have set *γ* = (*n* − 1)/(*n* − 1 + *αG*). If we then calculate *ϕ*_*r*_ − *ϕ*_*m*_ for an arbitrary resident strategy and a cooperative invader, *r*_*m*_ = 1 − *s*_*m*_ we find

5.21 $$\begin{array}{rl} \phi_{r}-\phi_{m} & =-(1-r_{r}-s_{r})\\ & \;\times \frac{\left(1-s_{m})((1-\gamma )s_{r}(B-Cs_{r})-C(1-s_{r}^{2})n\right)}{\left(1-s_{r})(n-s_{r}(s_{m}+s_{r}(n-1))\right)}. \end{array}$$

Equations (5.20) and (5.21) are identical except for initial factor *r*_*r*_ in equation (5.21) and −(1 − *r*_*r*_ − *s*_*r*_) in equation (5.21). And so any strategy that is not completely cooperative or completely non-cooperative can be invaded either by a cooperative or a non-cooperative strategy.

Next we must determine the stability of fully cooperative and fully non-cooperative strategies. First we note that any pair of fully cooperative strategies always cooperate with one another, and so can replace one another via neutral drift [33]. Similarly, any pair of fully non-cooperative strategies always defect against one another and can similarly replace one another via neutral drift. As a result there are no strictly Evolutionary Stable Strategies in this system, since invasions can always occur via drift. Nonetheless, fully cooperative and fully non-cooperative strategies may be *evolutionary robust*, meaning that they cannot be invaded other than by neutral drift [30].

In order to determine the conditions for fully cooperative strategies to be evolutionary robust, we look at the conditions for invasion against such a resident strategy, *r*_*r*_ = 1 − *s*_*r*_, by an arbitrary invader *r*_*m*_ < 1 − *s*_*m*_. Substituting from equation (5.12) we find

5.22 $$\begin{array}{rl} \phi_{r}-\phi_{m} & =-(1-s_{m}-r_{m})\\ & \;\times \frac{B(1-\gamma )s_{r}-C(n-s_{r}^{2}(n-(1-\gamma )))}{n-s_{r}(s_{m}+s_{r}(n-1))}, \end{array}$$

and the resident strategy can resist invasion provided

5.23 $$s_{r}>\frac{-B(1-\gamma )+\sqrt{{\left(B(1-\gamma )\right)}^{2}+4C^{2}n(n-(1-\gamma ))}}{2C(n-(1-\gamma ))}.$$

Similarly, for a fully non-cooperative invader, we look at the conditions for a resident strategy *r*_*r*_ = 0 to resist invasion against an invader *r*_*m*_ > 0. Substituting from equation (5.12) we find

5.24 $$\phi_{r}-\phi_{m}=r_{m}\times \frac{B(1-\gamma )s_{r}-C(n-s_{r}^{2}(n-(1-\gamma )))}{n-s_{r}(s_{m}+s_{r}(n-1))},$$

and the resident strategy can resist invasion provided

5.25 $$s_{r}<\frac{-B(1-\gamma )+\sqrt{{\left(B(1-\gamma )\right)}^{2}+4C^{2}n(n-(1-\gamma ))}}{2C(n-(1-\gamma ))}.$$

We can now calculate the proportion of cooperative and non-cooperative strategies that are evolutionary robust. Setting

5.26 $$\rho =1-\frac{-B(1-\gamma )+\sqrt{{\left(B(1-\gamma )\right)}^{2}+4C^{2}n(n-(1-\gamma ))}}{2C(n-(1-\gamma ))},$$

from equation (5.23) the probability that a randomly drown cooperative strategy is robust is *ρ*, while the probability that a randomly drawn non-cooperative strategy is robust is 1 − *ρ*. Taylor expanding equation (5.26) in 1/*n* yields the approximate expression for robustness given in the main text.

### (e) Evolutionary dynamics

Having characterized the evolutionary robust strategies associated with the system, we can also characterize the evolutionary dynamics. In particular, under the weak mutation limit with global mutations, in which new invading strategies enter a stereotype group and are either lost or go to fixation before a new invader arises, the long-term evolutionary dynamics consist of long periods of quasi-stable cooperative and non-cooperative strategies [33], which are slowly eroded by drift (see electronic supplementary material, figure S2). Under these dynamics the average rate of cooperation depends on the relative robustness of cooperative and non-cooperative strategies, given by *ρ* and 1 − *ρ* respectively with the probability that a given individual is willing to engage in cooperation is given by equation (2.2) [33] (see electronic supplementary material, section S1 for full details).

## Data Availability

Additional simulation results and analysis are provided in electronic supplementary material [58].
